# Current Practice of Fluid Maintenance and Replacement Therapy in Mechanically Ventilated Critically Ill Children: A European Survey

**DOI:** 10.3389/fped.2022.828637

**Published:** 2022-02-23

**Authors:** Ismail Arrahmani, Sarah A. Ingelse, Job B. M. van Woensel, Reinout A. Bem, Joris Lemson

**Affiliations:** ^1^Department of Pediatric Intensive Care, Emma Children's Hospital, Amsterdam University Medical Center, University of Amsterdam, Amsterdam, Netherlands; ^2^Department of Intensive Care, Radboud University Medical Center, Nijmegen, Netherlands

**Keywords:** fluid balance, edema, mechanical ventilation, children, pediatric intensive care unit

## Abstract

Appropriate fluid management in mechanically ventilated critically ill children remains an important challenge and topic of active discussion in pediatric intensive care medicine. An increasing number of studies show an association between a positive fluid balance or fluid overload and adverse outcomes. However, to date, no international consensus regarding fluid management or removal strategies exists. The aim of this study was to obtain more insight into the current clinical practice of fluid therapy in mechanically ventilated critically ill children. On behalf of the section of cardiovascular dynamics of the European Society of Pediatric and Neonatal Intensive Care (ESPNIC) we conducted an anonymous survey among pediatric intensive care unit (PICU) specialists in Europe regarding fluid overload and management. A total of 107 study participants responded to the survey. The vast majority of respondents considers fluid overload to be a common phenomenon in mechanically ventilated children and believes this complication is associated with adverse outcomes, such as mortality and duration of respiratory support. Yet, only 75% of the respondents administers a lower volume of fluids (reduction of 20% of normal intake) to mechanically ventilated critically ill children on admission. During PICU stay, a cumulative fluid balance of more than 5% is considered to be an indication to reduce fluid intake and start diuretic treatment in most respondents. Next to fluid balance calculation, the occurrence of peripheral and/or pulmonary edema (as assessed including by chest radiograph and lung ultrasound) was considered an important clinical sign of fluid overload entailing further therapeutic action. In conclusion, fluid overload in mechanically ventilated critically ill children is considered an important problem among PICU specialists, but there is great heterogeneity in the current clinical practice to avoid this complication. We identify a great need for further prospective and randomized investigation of the effects of (restrictive) fluid strategies in the PICU.

## Introduction

Increasing evidence shows that overzealous use of (intravenous) fluids in critically ill patients beyond the resuscitation phase is associated with adverse outcome (1–3). A recent systematic review and meta-analysis showed that fluid overload in critically ill children admitted to the pediatric intensive care unit (PICU) was associated with fewer ventilator free days, a higher risk of acute kidney injury and even an increased risk of mortality (4). To our knowledge, no clinical trials comparing liberal vs. restrictive fluid therapy strategies in critically ill children have been published.

Restricting the amount of fluids is a daily clinical challenge, particularly in critically ill children undergoing invasive mechanical ventilation. Specifically, because the administration of fluid is necessary to provide hemodynamic support and to ensure appropriate caloric/protein intake while at the same time it functions as a vehicle for drug delivery (fluid creep) (5, 6). As a result, a positive (cumulative) fluid balance and the formation of edema are very common in these children (3, 4, 7, 8). However, fluid maintenance strategies and the use of diuretic medications in the PICU environment may vary. More insight into the current clinical practice of fluid therapy in critically ill children is necessary. This information can be used to design and guide future trials that might lead to international consensus and evidence-based guidelines.

The main goal of this study was to gain insight into the current clinical practice and attitudes of PICU clinicians regarding fluid maintenance and replacement therapy in mechanically ventilated critically ill children.

## Methods

### Survey Design

This web-based, anonymous survey was designed using Surveymonkey^®^. The survey was composed using the contribution of all authors. In the process, besides email contact, we organized two discussion sessions and multiple dry runs. The conceptual survey questions were sent to an independent colleague with expertise regarding the subject, for review concerning clarity, relevance and topic coverage.

The survey was written in English and comprised of a total of 47 questions divided over eight sections (Demographic information, Statements regarding fluid management and overload, Monitoring fluid balance, Interventions, Nutrition and enteral feeding and Future studies). As stated in the survey (Supplementary Material) the questions focused on “general” invasive mechanically ventilated (expected duration >48 h) PICU patients, excluding post-transplant patients, post-cardiothoracic surgery patients and patients with pre-existing cardiac and kidney dysfunction prior to admission to PICU. The survey consisted of a combination of multiple-choice questions, Likert-scales and free text responses. The full questionnaire of this survey can be found as an online supplement to this article (Supplementary Material).

Participants were asked for consent at the start of the survey and were given the opportunity to leave comments and demographic information on a voluntary basis. A waiver from the local ethical committee for the distribution of the survey was obtained (W21-388). The survey was created and distributed following current available recommendations where possible and appropriate (9).

### Target Respondents and Survey Distribution

Only (fellow-) pediatric-intensivists and PICU nurse practitioners were asked to fill in the survey. Furthermore, it was also required for the participants to work in a European country. The survey invitation was distributed through the European Society of Pediatric Neonatal Intensive Care (ESPNIC) newsletter and by email personally directed to all members of the section of cardiovascular dynamics of ESPNIC. In the Netherlands, the survey was also distributed via email to the members of the Dutch Society of Pediatric Intensive Care. Finally, in order to increase the response rate, the survey was also distributed to other colleagues using the authors' personal contact list. Official email reminders were sent twice, at 1 and 3 months after the initial distribution of the survey. In addition, monthly reminders were sent out through social media platforms of ESPNIC (e.g., official ESPNIC Facebook page, twitter, LinkedIn).

### Data Collection

Data from the online survey were collected from November 2020 until April of 2021.

### Statistical Analysis

Statistical analysis was performed using IBM statistics SPSS v26.0. Data were analyzed with descriptive measures and presented as proportion, percentage and median (interquartile range, IQR). For each question, the total number of respondents may due to an incomplete survey response leading to missing answers. Therefore, we present the number of respondents per survey question throughout the paper. Data from Likert scales were enumerated as ordinal data ranging from 1 to 5, with 1 = strongly disagree and 5 = strongly agree.

## Results

### Demographic Information

**Figure 1:**
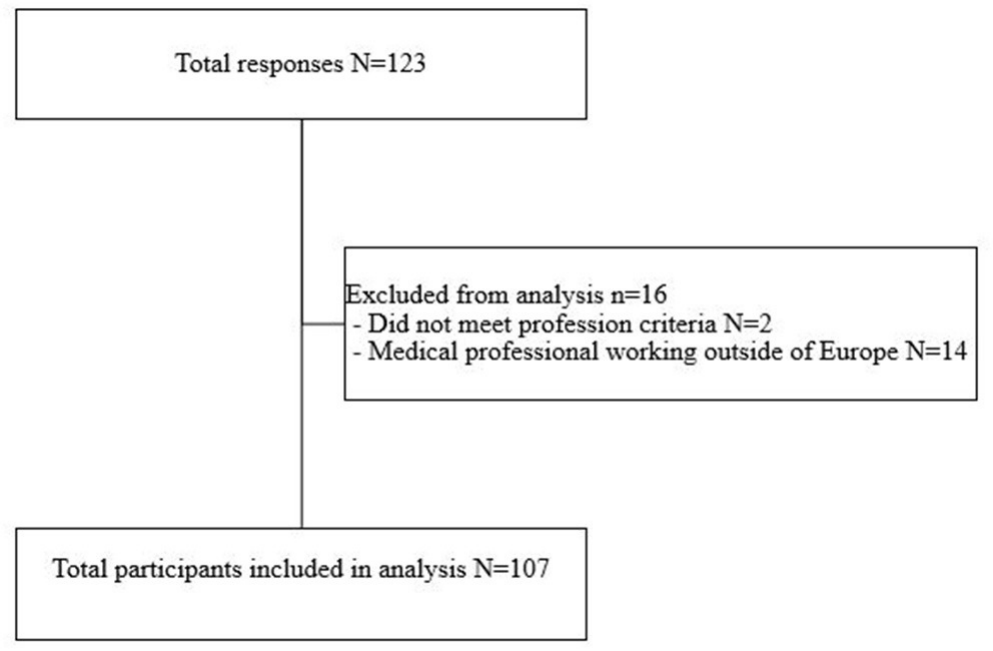
Inclusion of respondents.

After exclusion of those respondents that were not eligible to participate, a total of 107 respondents were included in the final analysis (Figure 1). 82/107 (76.6%) of the respondents completed all the mandatory questions of the survey. 55/107 (48.6%) of the respondents came from the Netherlands, 17/107 (15.9%) from Germany and 16/107 (15.0%) from the UK (Table 1).

**Table 1:** Demographics.

	**N (%)**
Total number of respondents after exclusion	107 (100)
**Country of PICU location**	
Netherlands	55 (48.6)
Germany	17 (15.9)
UK	16 (15)
Belgium	1 (0.9)
Czech Republic	1 (0.9)
France	2 (1.9)
Greece	2 (1.9)
Hungary	1 (0.9)
Italy	4 (3.7)
Norway	1 (0.9)
Spain	5 (4.7)
Switzerland	2 (1.9)
Ukraine	2 (1.9)
Macedonia	1 (0.9)
**Medical profession**	
Pediatric-intensivist	95 (88.5)
Fellow pediatric-intensivist	7 (6.5)
PICU nurse-practitioner	4 (3.7)
**Years of experience working in PICU**	
>10 years	65 (60.7)
5–10 years	16 (15.0)
0–5 years	26 (24.3)
**PICU facility**	
General PICU	49 (45.7)
Cardiac PICU	1 (0.9)
Mixed cardiac and general PICU	45 (42.1)
Mixed PICU-neonatology (N)ICU	12 (11.2)

As depicted in Table 1, 95/107 (88.5%) of the respondents were practicing pediatric-intensivists and had over more than 10 years (*N* = 65/107, 60.7%) of clinical experience working in a PICU. The respondents stated to work in a general-, cardiac-, or mixed PICU in 49/107 (45.7%), 1/107 (0.9%), and 45/107 (42.1%) of the cases, respectively. 12/107 (11.2%) reported to work in a combined PICU-neonatology (N)ICU.

### View on Fluid Management

**Figure 2:**
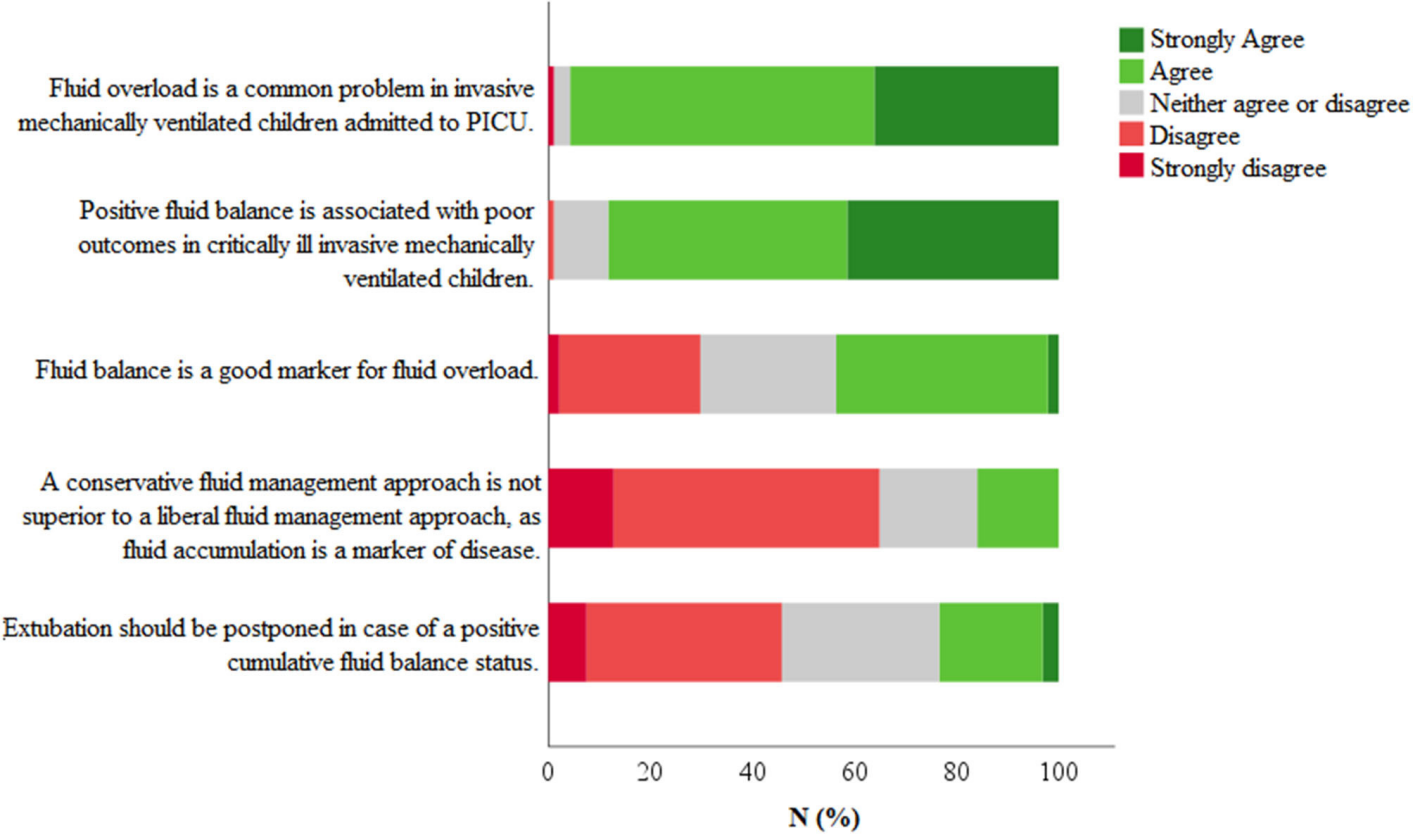
Statements regarding fluid management. Respondents were asked to give their opinion on several statements using a 5-point Likert scale (strongly disagree–strongly agree). Total number of respondents *N* = 82.

Respondents were asked to give their view on several statements concerning the topic fluid overload and management. As shown in Figure 2, 82/86 (95.7%) respondents considered fluid overload to be a common problem in invasive mechanically ventilated children admitted to the PICU. In addition, 76/86 (88.3%) respondents agreed or strongly agreed with the argument that positive fluid balance is associated with a poor outcome in these patients. In line with this, 56/86 (64.9%) respondents believed that a conservative fluid management approach will be beneficial. The results of all statements are shown in the Supplementary Figure 1.

### Determining Total Fluid Maintenance Requirement

In the survey, fluid maintenance was defined as all fluids administered during the course of mechanical ventilation, including medication, nutrition, fluid challenges. 57/86 (66.3%) of the respondents reported the presence of a local written protocol concerning total fluid management in invasive mechanically ventilated patients. In 55/86 (63.9%) of cases, the existing protocol was intended for all admitted patients, while in the other cases the protocol was primarily intended for a specific PICU population (e.g., post-cardiac surgery, post general surgery, septic patients).

55/86 (63.9%) of the respondents reported that they use the Holiday-Segar formula (4 ml/kg/h for the first 10 kg + 2 ml/kg/u for the second 10 kg + 1 ml/kg/u>20 kg) to determine the normal daily total fluid volume requirement in healthy children (non-critically ill, non-mechanically ventilated patients). For PICU patients at the start of invasive mechanical ventilation, 64/86 (74.4%) of the respondents reported to give less fluids (median (IQR) 20% (20–30)] based on the calculated normal daily total fluid volume. The remainder of the respondents 21/86 (24.4 %) stated to give the full 100% of calculated normal maintenance fluid.

Balanced crystalloid solutions were the preferred choice during intravenous fluid therapy followed by crystalloid solutions. A small minority [3/82 (3.7%)] preferred colloid solutions, such as albumin and hydroxyethyl starch.

Table 2 depicts the response concerning fluid resuscitation and hypovolemia. When asked about the way the respondents determined the need for fluid resuscitation in invasive mechanically ventilated children, 79/82 (96.3%) of the participants reported clinical signs (e.g., central capillary refill, color, peripheral temperature) as a marker for hypovolemic state. Heart rate, blood pressure, urine production and fluid responsiveness were also frequently (in >90% of the responses) considered as markers for a hypovolemic state.

**Table 2:** Fluid resuscitation and hypovolemia.

	**N (%)**
Total number of respondents	82 (100)
**How do you determine if your patient is in need of fluid resuscitation? (please check ALL that apply)**	
Based upon clinical signs (like refill, colour, peripheral temperature)	79 (96.3)
Based upon heart rate and or blood pressure	75 (91.5)
Based upon urine production	74 (90.2)
Based upon additional diagnostics (ultrasound, advanced hemodynamic monitoring, etc.)	60 (73.2)
Based upon laboratory diagnostics like urea	35 (42.7)
Based upon increased lactate level	66 (80.5)
Based upon a measure of fluid responsiveness	74 (90.2)
Other, please specify^*^	5 (6.1)
**In case of a hypovolemic state, what volume of fluid bolus do you typically give to an invasive mechanically ventilated child that is hemodynamically stable without cardiac disease?**	
None	3 (3.7)
5 ml/kg	8 (9.8)
10 ml/kg	59 (72.0)
15 ml/kg	1 (1.2)
20 ml/kg	11 (13.4)
**Do you determine fluid responsiveness before administering a fluid bolus**	
Always	9 (11.0)
Often	32 (39.0)
Sometimes	32 (39.0)
Rarely	7 (8.5)
Never	2 (2.4)
**If you determine fluid responsiveness, what method do you use most often? (please check ALL that apply)**	
N/A	4 (4.9)
Passive leg raising	32 (39.0)
Arterial pressure variations	44 (53.7)
Peak flow variations in aorta using ultrasound/Doppler	6 (7.3)
Mini fluid bolus	31 (37.8)
CVP	21 (25.6)
Diameter of the inferior vena cava using ultrasound	37 (45.1)
Liver compression	47 (57.3)
Other, please specify^**^	4 (4.9)
**If you deliver a fluid bolus as fluid resuscitation, how do you establish its beneficial effect? (please check ALL that apply)**	
An increase in blood pressure	57 (69.5)
A decrease in heart rate	80 (97.6)
An increase in urine production	64 (78.0)
An increase in cardiac output	29 (35.4)
Improved clinical signs	78 (95.1)
Improved NIRS measurement	17 (20.7)
Other, please specify^***^	5 (6.1)

* *Other: central venous oxygen saturation n = 2, passive leg raising test n = 1, based on pathophysiology n = 2*.

** *Other: heart rate changes n = 2, PiCCO n = 2*.

*** *Other: decrease in pulse pressure variation n = 2, improved serum lactate/base excess n=2, Not specified n = 1*.

*CVP, central venous pressure; NIRS, Near-infrared spectroscopy; PiCCO, Pulse index Continuous Cardiac Output*.

When administering a fluid bolus, 59/82 (72.0%) of the participants preferred a fluid bolus of 10 ml/kg in invasive mechanically children without signs of cardiac failure. 11/82 (13.4%) of the respondents reported the use of a fluid bolus of 20 ml/kg.

### Monitoring Fluid Overload and Interventions

The choice of fluid overload monitoring in invasively mechanically ventilated children was divided between the respondents of the survey. More than one third of the respondents claimed that NET or cumulative fluid balance was monitored hourly, followed by every six (*N* = 21/82, 25.6%) or eight (*N* = 14/82, 17.1%) hours.

**Figure 3:**
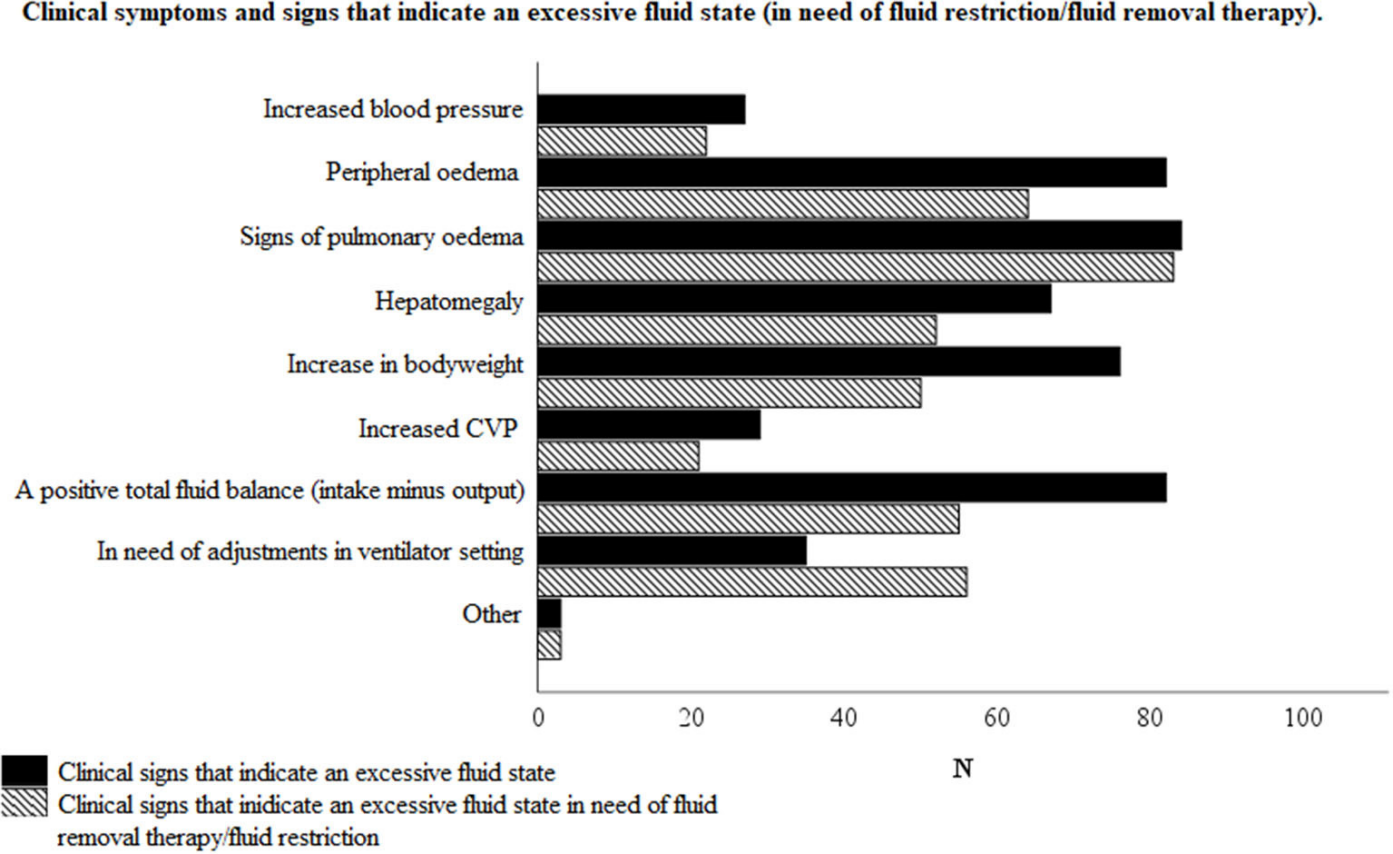
Clinical symptoms and signs that indicate an excessive fluid state with or without the need of fluid removal therapy and/or fluid restriction, according to the respondents. Total number of respondents *N* = 82.

Figure 3 illustrates the reported clinical signs of an excessive fluid state (as considered to be in need of fluid removal therapy). Peripheral edema, signs of pulmonary edema and a positive total fluid balance, as clinical signs of an excessive fluid state, were reported in 77/82 (93.9%), 79/82 (96.3%), and 76/82 (92.7%) of the responses, respectively. On the other hand, peripheral edema, signs of pulmonary edema and a positive total fluid balance, as clinical signs addressing the need for fluid removal therapy and /or fluid restriction, were reported in 61/82 (74.4%), 78/82 (95.1%), and 51/82 (62.2%) of cases, respectively.

Table 3 depicts tools (clinical, radiological and/or laboratory) used for diagnosing fluid overload. For this purpose, laboratory findings (e.g., urea, creatinine, NT-pro BNP), chest-X-ray and lung ultrasound were reported to be utilized in 60/82 (73.1%), 57/82 (69.5%), and 42/82 (51.2%) of the responses, respectively.

**Table 3:** Tools (clinical, radiological and/or laboratory) used regularly diagnosing fluid overload.

	**N (%)**
Total number of respondents	82 (100)
Lung ultrasound	42 (51.2)
Cardiac ultrasound	40 (48.8)
Chest-X-ray	57 (69.5)
PiCCO (transpulmonary thermodilution)	9 (11.0)
Laboratory findings (e.g., ureum, creatinin, NT-proBNP)	60 (73.1)
Other^*^	13 (17.1)

* *Other: based on clinical/physical examination of the patient (N = 10, 13.0%), Fluid overload in percentage (N = 1, 1.2%)*.

*PiCCO, Pulse index Continuous Cardiac Output*.

As presented in Table 4, there was a considerable variation in clinical practice regarding the threshold of positive cumulative fluid balance at which fluid management adjustments were accomplished. While a small majority (51.2%) of the respondents would change the fluid management above 5% positive cumulative fluid balance, 28% of the clinicians reported to make adjustments only when clinical signs of fluid overload were observed. Decreasing the amount of maintenance fluidas was reported to be the primary intervention in case of an excessive fluid state of the patient by 67/82 (81.7%) respondents. Starting diuretic drug therapy as the initial approach was reported by 72/82 (87.8%) of the cases. Initial diuretics prescribed included intermitted loop diuretics (reported by 73/82 (89.0%) of the respondents), potassium sparing drugs in 36/82 (43.9%) of the cases, and 20/82 (24.4%) of the respondents preferred continuous loop diuretic drip infusion. Thiazide diuretics were reported to be used in only 9/82 (11.0%) of the responders. Early initiation of continuous renal replacement therapy (CRRT) was reported to be the initial approach in 4/82 (4.8%) of the cases while 55/82 (67.1%) of the respondents indicated that CRRT is never or rarely used to manage fluid overload as the sole indication. In 27/82 (32.9%) of the cases, CRRT is reported to be used as treatment for fluid overload as the sole indication.

**Table 4:** Fluid balance and indication of fluid removal therapy.

	**N (%)**
Total number of respondents	82 (100)
**What cumulative fluid balance (%) (since admission in PICU AND start of mechanical ventilation) is a reason for making fluid management changes?**	
Even fluid balance, one should strive for a negative fluid balance	14 (17.1)
0–5% fluid positive	3 (3.7)
5%−10% fluid positive	29 (35.4)
10%−15% fluid positive	22 (13.4)
15%−20% fluid positive	2 (2.4)
Other^*^	23 (28.0)
**What is the preferred initial drug therapy used for fluid removal in case of positive fluid balance or signs of fluid overload in invasive mechanically ventilated PICU patients?**	
Intermittent loop diuretics	73 (89.0)
Continuous loop diuretics drip infusion	20 (24.4)
Thiazide diuretic (e.g., hydrochlorothiazide)	9 (11.0)
Potassium sparing (e.g., spironolactone)	36 (43.9)
I do not use diuretics for fluid removal therapy	0 (0.0)
Other	0 (0.0)
**In case of changing fluid management, due to positive fluid balance or clinical signs of fluid overload, what is the initial intervention used for fluid removal?**	
Lowering fluid maintenance	67 (81.7)
Avoidance of maintenance fluid and minimization of drug diluents	40 (48.8)
Start diuretic drug therapy	72 (87.8)
Early start of renal replacement therapy	4 (4.9)
Watchful waiting	3 (3.7)
No intervention	0 (0.0)
Other^**^	2 (2.4)
**How often is continuous renal replacement treatment (CRRT) used to manage fluid overload as the sole indication?**	
Always	0 (0.0)
Usually	6 (5.6)
Sometimes	21 (19.6)
Rarely	41 (38.3)
Never	14 (13.1)

* *Other: Changes in fluid management only in combination with clinical signs (N = 23, 28.0%)*.

** *Other: switch to enteral feeding (N = 1, 1.2%), not further specified (N = 1, 1.2%)*.

### Nutrition and Enteral Feeding

In order to maintain an acceptable fluid balance, 37/82 (45.1%) of the survey respondents (strongly) agreed that energy requirements of the patients can be decreased. On the other hand, 32/82 (39.0%) (strongly) disagreed with this statement. Enteral administration of fluid by feeding (non-resuscitation) was considered the preferred method in 75/82 (91.5%) of the cases.

### Future Trial Design

**Figure 4:**
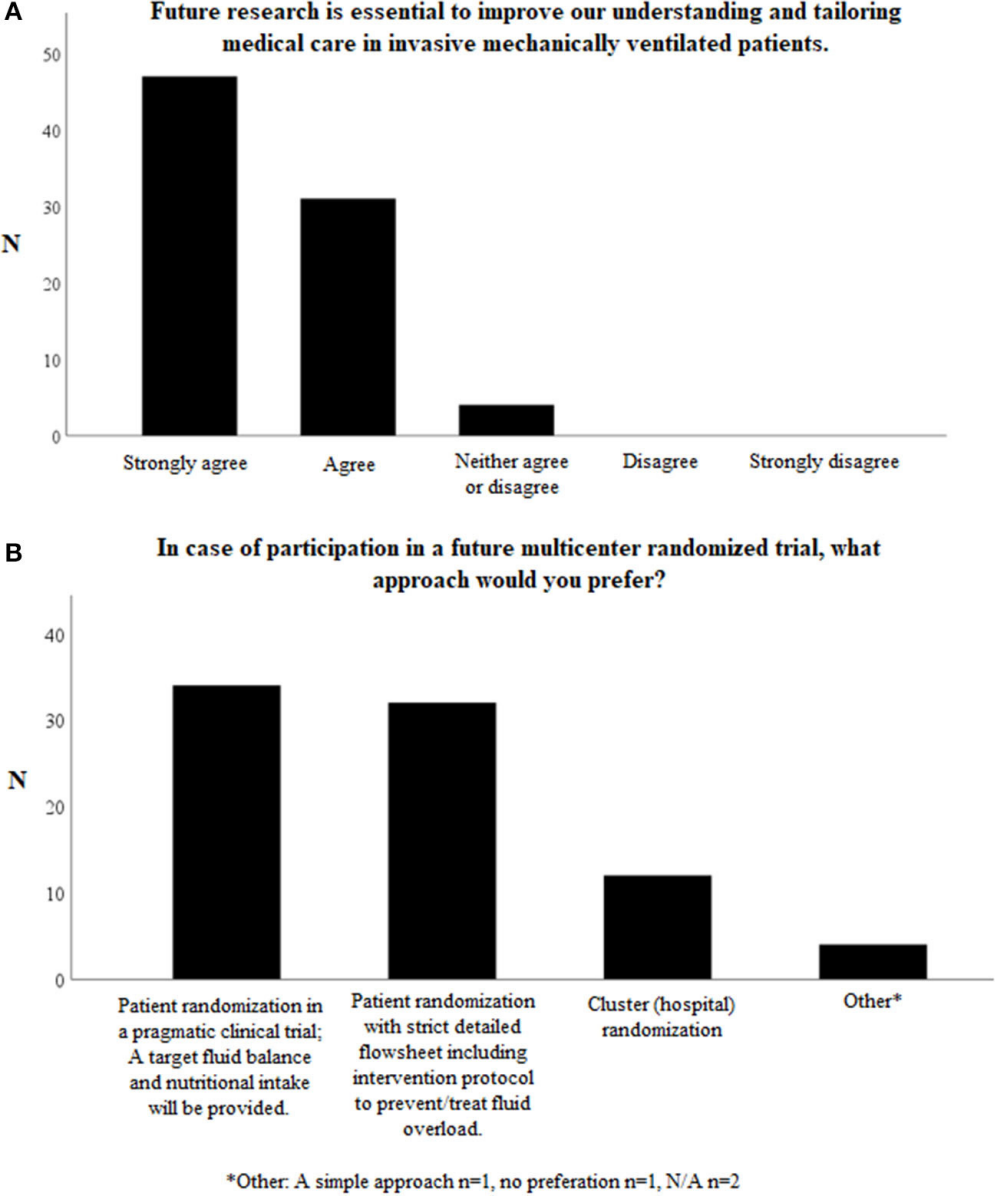
Questions on possible future studies on fluid management in invasively mechanically children. Total number of respondents *N* = 82. **(A)** Further research in fluid management is essential to improve our understanding and tailoring medical care in invasive mechanically ventilated patients. **(B)** In case of participation in a future multicenter randomized trial, what approach would you prefer? *Other. A simple approach *n* = 1, no preferation *n* = 1, N/A *n* = 2.

When asked whether future research in fluid management is essential to improve our understanding and tailoring medical care in invasive mechanically ventilated patients in the PICU, 78/82 (95.1%) respondents agreed or strongly agreed with the statement. None of the respondents disagreed or strongly disagreed (Figure 4A).

There is also a high willingness (*N* = 71/82, 86.6%) among participants to include patients in a future clinical trial investigating possible benefits of a conservative fluid approach. Some respondents who answered “no” believe there is already enough evidence to justify a conservative fluid management approach in invasive mechanically ventilated children. The other respondents argued allocating to a “liberal fluid management” arm would not be ethical, as a conservative approach has already been integrated in their current fluid management protocol.

When it comes to the preferred trial design, 34/82 (41.5%) of the respondents preferred a pragmatic clinical trial, whereas 32/82 (39.0%) preferred patient allocation to strict, detailed intervention protocols (Figure 4B).

The duration of mechanical ventilation was often reported as an important primary outcome of a future clinical study (*N* = 59/75, 78.7%). Other possible important primary outcomes, reported by respondents, were: PICU length of stay (*N* = 35/75, 46.7%), mortality (*N* = 20/75, 26.7%) and renal failure/ need for renal replacement therapy (*N* = 17/75, 22.7%).

## Discussion

The primary aim of this study was to obtain information regarding daily clinical practice and the opinion of clinicians concerning fluid maintenance and replacement therapy in critically ill children undergoing invasive mechanical ventilation. The results show that in the opinion of many PICU specialists, fluid overload is a serious problem and a possible threat to these patients. However, there seems to be no clear agreement in the recognition, prevention or treatment of fluid overload in the PICU.

Maintaining an optimal fluid status in critically ill patients is one of the challenging aspects of (P)ICU care. During critical illness, the blood circulation can be compromised due to several factors, including a (widespread) pro-inflammatory response. Inflammation may cause capillary leakage, with subsequent hypovolemia, and a reduced cardiac function, both leading to circulatory insufficiency. Invasive mechanical ventilation itself can also reduce the cardiac output by increasing the afterload, while also decreasing the preload of the right ventricle. In these circumstances fluid loading can be lifesaving, and is therefore recommended in the acute phase of severe disease states like septic shock (10, 11). In contrast, fluid overload my also develop as a result of overzealous fluid administration and/or a continuous high fluid intake in combination with a concomitant inappropriate production of ADH (SIADH) or kidney failure. This often leads to the accumulation of extravascular fluid, further aggravated by degradation of the glycocalyx, culminating in the formation of edema in several tissues including the lung (7, 12, 13). The tissue edema, which may co-exist with both hyper- and hypovolemic intravascular volume, is considered to be the main cause of adverse effects of fluid overload. This could explain the consistent finding that positive cumulative fluid balance is associated with poor outcomes in both critically ill adults and children (3, 7, 14–20).

The theoretical concept mentioned above, has clearly been embraced by the critical care community, fueling a large number of studies on fluid management over the last years. In our survey, the vast majority of respondents indeed considered fluid overload to be a common problem in the PICU, associated with a poor outcome (Figure 2). This is on par with published reports from both pediatric and adult intensive care medicine (17, 18).

Considering fluid replacement, the majority of respondents used a volume of 10 ml/kg as a fluid bolus. This is in conjunction with current guidelines and studies (11, 21) although 20 ml/kg is still used by some. Fluid therapy was guided most frequently by clinical signs and symptoms, although research has shown that these do not always predict fluid responsiveness in a reliably way (22, 23). Fluid responsiveness, defined as an increase in cardiac output as a result of fluid loading, was sometimes determined using various methods. Unfortunately, these methods have not been fully validated in (smaller) children, and the effect of fluid loading is often not tested against a reliable effect parameter like cardiac output (22). This can be a result of the sparse use of invasive tools for hemodynamic monitoring, such as central venous pressure or pulmonary artery catheters, in children as compared to adults. Therefore, in contrast to the adult ICU, fluid resuscitation is not always performed based upon valid parameters and might contribute to fluid overload (23, 24). Future research needs to identify more accurate parameters for guiding fluid resuscitation in critically ill children in order to prevent fluid overload.

The majority of respondents monitor the fluid balance and possible signs of fluid overload regularly. However, there was large variability in the clinical practice regarding the threshold of a positive cumulative fluid balance at which adjustments to the fluid management strategy were applied. A close majority considered an increase of more than 5% in cumulative fluid balance a reason for intervention. Advanced diagnostics, when used, were diverse, ranging from chest x-ray to laboratory tests. A recent systemic review showed that positive cumulative fluid balance is indeed a risk factor for increased mortality in adult ICU patients (18). The increasing body of evidence from both adult and pediatric studies displaying a consistent positive association between fluid overload and adverse outcomes seems to convince many PICU clinicians that fluid overload is a threat to critically ill children.

Although several studies have shown that a positive cumulative fluid balance is associated with a worse outcome as early as day 1 of PICU admission, in a recent study among children with pediatric ARDS this association was only apparent after day 4 of disease progression (4, 7, 14). Also, in adults, a negative cumulative fluid balance at day 4 of acute lung injury was associated with significantly lower mortality (25). Van Regenmortel et al. (26) aimed to quantify all fluid sources and assess fluid creep in adult ICU-patients. Maintenance and replacement fluids accounted for 24.7% of the total daily fluid volume, whereas fluid creep represented 32.6% of the total daily fluid volume. Therefore, to limit fluid intake, both fluid maintenance and creep during drug delivery needs to be addressed by clinicians. In our survey, the majority of participants reported to give less than 80% of the Holliday-Segar formula, but a protocol was present in only 64% of the cases. This can be explained by the absence of an international guideline concerning continuous fluid management in critically ill (ventilated) children.

Loop diuretics were mentioned in our survey as the predominant first line drug intervention. However, there was no uniform strategy to counteract pending fluid overload. Lowering maintenance fluids, diuretics or avoidance of maintenance fluid and minimizing drug diluents were all mentioned almost equally. Unlike to the adult ICU literature, no guidelines for de-resuscitation or protocols for diuresis after the resuscitation phase in critically ill children have been published to our knowledge (16, 27). When loop diuretics do not increase urine production to the desired level, CRRT is the most efficient way to treat fluid overload. CRRT exploited solely for treating or preventing fluid overload is regarded as a relative indication, and timing and indications are still under debate (28). However, a recent survey showed that during pediatric extracorporeal life support (ECLS) an increasing number of centers use CRRT as a tool to prevent or treat fluid overload (29). Studies in adults have shown improved oxygenation indices and shorter ICU length of stay with a restrictive fluid strategy, however no survival benefit has been shown thus far (20). Trials for fluid therapy in children have been proposed for many years but still not accomplished (30). The general opinion seems to be that both hemodynamic and respiratory therapy needs to be personalized implying that a one-size-fits all strategy will not be realistic (20, 31).

## Future Perspectives

The current lack of randomized clinical trials hampers the development of widely accepted international guidelines. In terms of future research regarding fluid overload in the PICU, some problems have to be overcome.

First, current fluid strategies among participating centers will likely vary to a great extent and should thus be aligned. In particular, determining the volume of fluids in the liberal treatment arm might pose a difficulty, considering that centers, in which restriction of maintenance fluids is already common practice, may raise ethical concerns.

Second, there are questions related to timing, type and aggressiveness of interventions (e.g., fluid restriction, diuretic medication, CRRT) in a conservative fluid treatment arm complicating trial design (32). The ability to actually avoid fluid accumulation in critically ill children may be limited (5). This might require aggressive fluid restriction protocols directly following the resuscitation phase. As a result, incorporation of a broad set of safety parameters and long-term outcome measurements are therefore imperative.

Third, obtaining an appropriate sample size may be challenging. The adult ARDS FACTT-study enrolled 1,000 patients and had enough power to detect a 10% absolute reduction (from 31 to 21%) in mortality (1). In less prevalent pediatric ARDS, mortality is about half at 17.1% (SD 38.7), and thus a 5% absolute reduction in death rate would necessitate a sample size of more than 1,500 patients (12). This sample size would rise to above 6,000 patients for a trial including all critically ill children (also non-ARDS), in order to be able to detect a significant reduction in mortality, providing the case fatality of 3.1% (7). Such numbers are unrealistic for pediatric critical care research, and thus focus should lie on alternative primary outcomes such as duration of mechanical ventilation, as well as effects on a specific sub-groups of PICU patients (5).

Finally, the results of this survey show a high willingness among PICU clinicians to participate in a clinical trial in which children are to be randomized into a liberal vs. a conservative fluid strategy. Without such a trial, an evidenced based guideline cannot be accomplished.

There are several limitations to this study. First, the response did not follow an even distribution from the various European countries. There could be a bias as the majority of the respondents originated from the Netherlands, Germany and the UK. Second, the number of respondents was not very high and poses the question if the results reflect the opinion of the majority of European pediatric intensivists. Third, the results reflect the individual opinion of clinicians and not institutional or departmental policy. Fourth, intensivists that consider fluid overload a problem may have been more motivated to participate in the survey causing bias.

## Conclusion

Pediatric intensivists consider fluid overload an important problem and a possible threat to invasive mechanically ventilated critically ill children. However, currently there seems to be no agreement on fluid-sparing strategies and interventions to avoid this complication. Therefore, clinical trials that address prevention and treatment of fluid overload in mechanically ventilated children in the PICU are highly needed.

## Data Availability Statement

The original contributions presented in the study are included in the article/Supplementary Material, further inquiries can be directed to the corresponding author.

## Ethics Statement

The studies involving human participants were reviewed and approved by Amsterdam University Medical Center. The Ethics Committee waived the requirement of written informed consent for participation.

## Author Contributions

IA: study inception, survey design and conduction, analysis, and report writing. SI and JW: survey design and revision of the report. RB and JL: study inception, survey design, analysis, and report writing. All authors contributed to the article and approved the submitted version.

## Conflict of Interest

The authors declare that the research was conducted in the absence of any commercial or financial relationships that could be construed as a potential conflict of interest.

## Publisher's Note

All claims expressed in this article are solely those of the authors and do not necessarily represent those of their affiliated organizations, or those of the publisher, the editors and the reviewers. Any product that may be evaluated in this article, or claim that may be made by its manufacturer, is not guaranteed or endorsed by the publisher.
