# Supplementary figures and images for: Current Practice of Fluid Maintenance and Replacement Therapy in Mechanically Ventilated Critically Ill Children: A European Survey
Source: Front Pediatr. 2022 Feb 23;10:828637. doi: 10.3389/fped.2022.828637 (PMC8906881; doi:10.3389/fped.2022.828637)

| 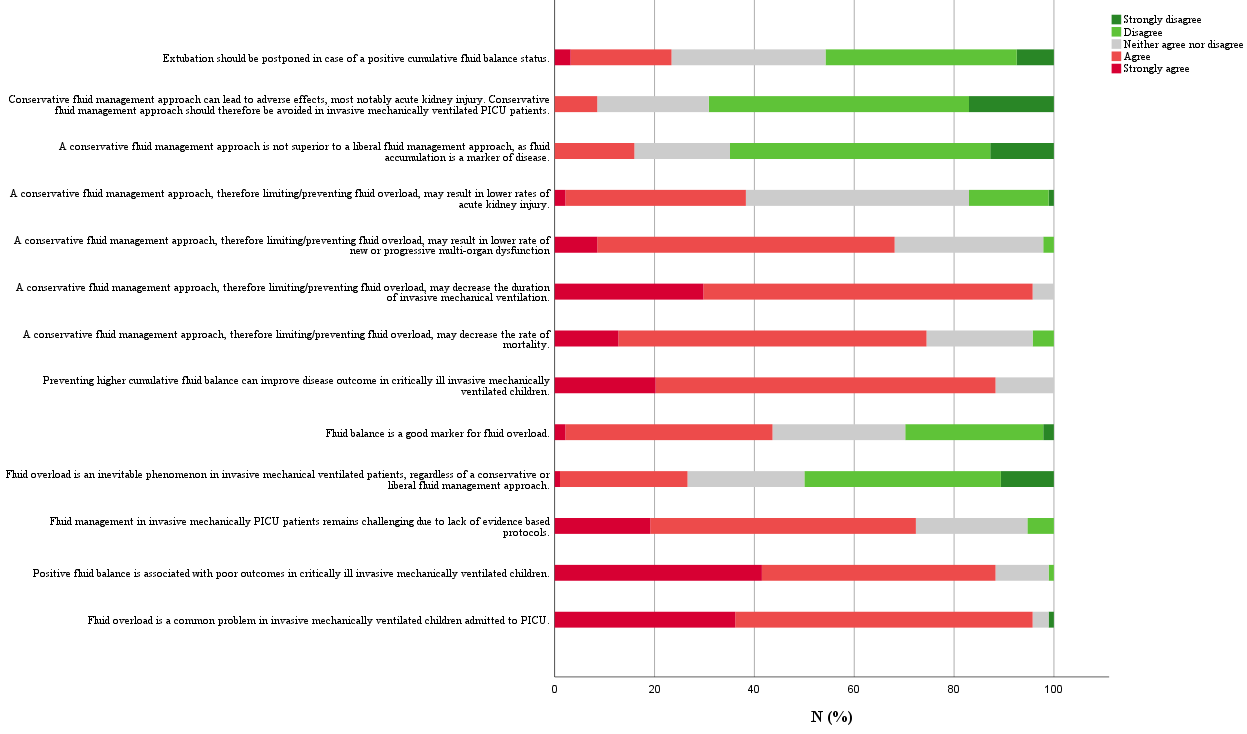 |
| --- |
| **Supplemental Figure 1| views on fluid management.** Total number of respondents N=82. |

Supplement: Supplementary file 1 [file Table_1.DOCX]
