# Supplementary material for: Current Practice of Fluid Maintenance and Replacement Therapy in Mechanically Ventilated Critically Ill Children: A European Survey
Source: Front Pediatr. 2022 Feb 23;10:828637. doi: 10.3389/fped.2022.828637 (PMC8906881; doi:10.3389/fped.2022.828637)
Supplement: Supplementary file 2 [file Table_2.DOCX]

| **Supplemental Table 1 \| Important features future clinical trial** | | |
| --- | --- | --- |
|  | | *N (%)* |
| Total number of respondents | | 75 (100) |
| What is, in your opinion, an important primary outcome regarding fluid management therapy in invasive mechanically ventilated PICU patients? | | |
| Duration of mechanical ventilation/30-day free mechanical ventilation days  Weaning time/failure  PICU length of stay  Mortality  Renal failure/ need for RRT  Delirium  Comorbidity not further specified  Fluid balance  Other* | 59 (78.7)  3 (4.0)  35 (46.7)  20 (26.7)  17 (22.7)  2 (2.7)  3 (4.0)  3 (4.0)  8 (10.7) |  |
| Should a future trial include a specific patient category (e.g. only invasive mechanically ventilated patients with respiratory tract infection). | | |
| Total number of respondents  Yes  No  Other** | 73 (100)  38 (52.1)  32 (43.8)  3 (4.1) |  |
| *Other: Appropriate management n=1, do not know n=1, inflammatory markers n=1, secondary infection n=1, costs n=1, N/A n=1, Timing of introduction of low caloric input TPN n=1, Days of nutrition Target achieved n=1, Long-term Lung function n=1.  **Other: N/A n=1, Difficult question n=1, is a possibility n=1. | | |
