# Supplementary material for: Current Practice of Fluid Maintenance and Replacement Therapy in Mechanically Ventilated Critically Ill Children: A European Survey
Source: Front Pediatr. 2022 Feb 23;10:828637. doi: 10.3389/fped.2022.828637 (PMC8906881; doi:10.3389/fped.2022.828637)
Supplement: Supplementary file 3 [file Data_Sheet_1.PDF]

Survey Total Fluid Management in Invasive Mechanically Ventilated Children

1. Introduction

Dear colleagues,

To date, fluid management in mechanically ventilated critically ill children remains an important topic of discussion. Several studies have demonstrated the association between positive fluid balance and adverse outcomes such as increased morbidity, and even mortality in critically ill patients. However, existing literature in the paediatric intensive care population is scarce. To our knowledge, no international guideline or consensus exists regarding fluid management in invasive mechanically ventilated children admitted to the PICU.

The purpose of this survey is to identify the common practice regarding total fluid management (i.e. including all fluids given like nutrition, medication, fluid challenges etc.) in invasive mechanically ventilated PICU patients. Furthermore, with this survey, we endeavour to understand the perspectives of paediatric critical care physicians in Europe regarding this subject. Your perspective on this subject will help to inform the design of a future clinical trial in this topic area. The results of this survey will be published as an European survey.

All answers given will be anonymously processed. By entering this survey, you are consenting with participation with this study. We kindly ask you to answer all the questions. The survey should require no more than 15 minutes to complete. This survey contains both open and multiple-choice questions. For the multiple-choice questions, we ask you to select the closest answer reflecting your common practice/opinion. We thank you for your time and effort spending in this survey.

For any technical questions and/or suggestions about this survey, please contact Ismail Arrahmani MD at: i.arrahmani@amsterdamumc.nl. For other general questions regarding the medical topic, please contact, Joris Lemson MD PhD at: joris.lemson@radboudumc.nl.

Kind regards,

Joris Lemson MD PhD, pediatric intensive care, Radboud university medical center

Reinout Bem MD PhD, paediatric intensive care, Emma Children's Hospital Amsterdam UMC

Ismail Arrahmani MD, paediatric intensive care, Emma Children's Hospital Amsterdam UMC

The section of cardiovascular dynamics of ESPNIC

This is an initiative of the Dutch PICU research network by;

Job van Woensel MD PhD paediatric intensive care, Emma Children's Hospital Amsterdam UMC

Joris Lemson MD PhD, pediatric intensive care, Radboud university medical center

Reinout Bem MD PhD, paediatric intensive care, Emma Children's Hospital Amsterdam UMC

Saranke Ingelse MD PhD, paediatric intensive care, Emma Children's Hospital Amsterdam UMC

* 1. Would you like to participate in this survey study?

- Yes
- No

* 2. What is your position in the PICU?

- Paediatric-intensivist/paediatric critical care physician
- Fellow paediatric-intensivist/ paediatric critical care physician
- PICU Physician assistant
- PICU Nurse Practitioner
- Other (please specify)

Survey Total Fluid Management in Invasive Mechanically Ventilated Children

2. Demographic information

Please provide us with some information about yourself and your workplace.

* 3. In what country do you work?

* 4. How many years have you been working (if applicable after completing training as a paediatric-intensivist/paediatric critical care physician) in the PICU?

- 0-5 years
- 5-10 years
- 10-15 years
- >15 years

* 5. What is approximately, in total, the number of admissions in your unit per year?

- 0 – 500
- 500 – 1000
- 1000 – 1500
- > 1500
- I do not know the anwser

* 6. In what type of PICU do you work in?

- General PICU
- Mixed cardiac and general PICU
- Cardiac PICU
- Mixed PICU-NICU
- Other (please specify)

* 7. What type of surgical patients are admitted to your PICU? (please check ALL that apply)

- Post-cardiothoracic surgery
- Post-transplantation surgery (heart, liver, kidney)
- Post-general surgery patients (including GE-surgery)
- Other (please specify)

Survey Total Fluid Management in Invasive Mechanically Ventilated Children

3. Questions about your views

* 8. The following questions/statements refer to your view and beliefs on fluid management.

	Strongly disagree	Disagree	Neither agree nor disagree	Agree	Strongly agree
Fluid overload is a common problem in invasive mechanically ventilated children admitted to PICU.	○	○	○	○	○
Positive fluid balance is associated with poor outcomes in critically ill invasive mechanically ventilated children.	○	○	○	○	○

	Strongly disagree	Disagree	Neither agree nor disagree	Agree	Strongly agree
Fluid management in invasive mechanically PICU patients remains challenging due to lack of evidence based protocols.					
Fluid overload is an inevitable phenomenon in invasive mechanical ventilated patients, regardless of a conservative or liberal fluid management approach.					
Fluid balance is a good marker for fluid overload.					
Preventing higher cumulative fluid balance can improve disease outcome in critically ill invasive mechanically ventilated children.					
A conservative fluid management approach, therefore limiting/preventing fluid overload, may decrease the rate of mortality.					
A conservative fluid management approach, therefore limiting/preventing fluid overload, may decrease the duration of invasive mechanical ventilation.					
A conservative fluid management approach, therefore limiting/preventing fluid overload, may result in lower rate of new or progressive multi-organ dysfunction					
A conservative fluid management approach, therefore limiting/preventing fluid overload, may result in lower rates of acute kidney injury.					

	Strongly disagree	Disagree	Neither agree nor disagree	Agree	Strongly agree
A conservative fluid management approach is not superior to a liberal fluid management approach, as fluid accumulation is a marker of disease.	◯	◯	◯	◯	◯
Conservative fluid management approach can lead to adverse effects, most notably acute kidney injury. Conservative fluid management approach should therefore be avoided in invasive mechanically ventilated PICU patients.	◯	◯	◯	◯	◯
Extubation should be postponed in case of a positive cumulative fluid balance status.	◯	◯	◯	◯	◯

Survey Total Fluid Management in Invasive Mechanically Ventilated Children

4. Determining total fluid requirement

The following questions refer to methods of determining fluid requirements (including all fluids given like medication, nutrion, fluid challenges etc.) of invasive mechanically ventilated PICU patients. Fill in the answers as it applies for the ''general" invasive mechanically ventilated (expectant duration >48hrs) PICU patients. *Excluding post-transplant patients, post-cardiothoracic surgery patients and patients with pre-existing cardiac and kidney dysfunction prior to admission to PICU.*

* 9. Is there a written protocol in your PICU regarding total fluid volume (including all fluids given like medication, nutrion, fluid challenges etc.) and fluid blance in invasive mechanically ventilated patients?

◯ Yes

◯ No

* 10. For which patient category is your protocol regarding fluid management (non- resuscitation) primary intended? (please check ALL that apply)

- [ ] All admitted PICU patients including non-mechanically ventilated
- [ ] Mechanically ventilated patients
- [ ] Post-cardiothoracic surgery
- [ ] Other (please specify)
- [ ] Post-transplantation surgery (heart, liver, kidney)
- [ ] Post-general surgery patients (including GE-surgery)
- [ ] Sepsis/Infectious disease

* 11. What calculation do you use to determine the ***normal*** daily total fluid volume (including additional fluid requirement like nutrition and medication) in non-critically ill, non-mechanically ventilated children? (please check ALL that apply)

- [ ] 1 month to < 1 year of age: 150ml/kg/day for children
- [ ] > 1 year of age: Conform ‘normal’ maintenance fluid based on the 4-2-1 rule or Holliday Segar formula (4ml/kg/hr for the first 10kg + 2ml/kg/u for the second 10kg + 1ml/kg/u>20kg))
- [ ] Conform ‘normal’ maintenance fluid based on the 4-2-1 rule or Holliday Segar formula (4ml/kg/hr for the first 10kg + 2ml/kg/u for the second 10kg + 1ml/kg/u>20kg) **for all ages**
- [ ] Conform ‘normal’ maintenance fluid based on body surface area method (1500-2000ml/m3/day)
- [ ] Other (please specify)

* 12. At start of mechanical ventilation, what rate/percentage do you choose for the daily total maintenance fluid volume (including all fluids given like medication, nutrition, fluid challenges etc.) in PICU patients with expected mechanical ventilated duration >48 hrs?

- ( ) 100% maintenance as calculated with the formula of the previous question
- ( ) More than the calculated rate based on the formula of the previous question.
- ( ) Less than the calculated rate based on the formula of the previous question?

* 13. If your anwser to Q14 was **more** than the calculated rate, how much more fluids do you administer (please give your answer in % or ml/kg/day)? (Fill in N/A if this question does not apply to you)

* 14. If your anwser to Q14 was **less** than the calculated rate, approximately how much less fluids do you administer (please give your answer % or ml/kg/day)? (Fill in N/A if this question does not apply to you)

* 15. At time of admission to the PICU what weight of patients is used for calculating the total maintenance fluid volume? (please check ALL that apply)

- [ ] All PICU patients are weighed at time of admission
- [ ] Ideal body weight (growth chart)
- [ ] The most recent weight, if noted is used
- [ ] Weight is not used
- [ ] Weight is estimated with the following formula: 2.5 x age + 8 in children >1 year.
- [ ] Other (please specify)

* 16. How often are mechanically ventilated patients, within the standard care, weighed during admission in the PICU?

- ○ Once a day
- ○ 3 times a week
- ○ Once a week
- ○ Other (please specify)

Survey Total Fluid Management in Invasive Mechanically Ventilated Children

5. Monitoring of fluid balance in mechanically ventilated PICU patients

Fill in the answers as it applies for the ‘’general” invasive mechanically ventilated (expected duration of > 48 hrs) PICU patients. ***Excluding post-transplant patients, post-cardiothoracic surgery patient and patients with pre-existing cardiac and kidney dysfunction prior to admission to PICU.***

* 17. When starting maintenance intravenous fluid, what type of solution is mostly used?

- ○ Crystalloid Solutions
- ○ Balanced crystalloid solutions
- ○ Colloid solutions
- ○ Other (please specify)

* 18. If your answer to Q19 was crystalloid solutions, what type of **crystalloid solution** is mostly used?

- ◯ 0.9% Saline (normal saline)
- ◯ 0.45% Saline (half saline)
- ◯ Glucose 5%
- ◯ Glucose 10%
- ◯ Glucose 5%, 0.9% Saline
- ◯ Glucose 5%, 0.45% Saline
- ◯ Glucose 2.5%, 0.45% Saline
- ◯ N/A
- ◯ Other (please specify)

19. If your answer to Q19 was balanced crystalloid solutions, what type of **balanced crystalloid solution** is mostly used?

- ◯ Ringer’s Lactate
- ◯ Sterofundin
- ◯ Plasma-Lyte A
- ◯ N/A
- ◯ Other (please specify)

20. If your answer to Q19 was colloid solutions, what type of **colloid solution** is mostly used?

- ◯ Albumin 5%
- ◯ Albumin 20%
- ◯ Hydroxyethyl starch (also Voluven, Volulyte and Hespan)
- ◯ N/A
- ◯ Other (please specify)

* 21. How is urine output usually measured in invasive mechanically ventilated childred?

- ◯ All invasive mechanically ventilated PICU patients have a urine catheter to monitor urine output.
- ◯ Urine output is measured by weighing diapers, unless a urine catheter is in place.
- ◯ Other (please specify)

* 22. How often is the NET or cumulative fluid balance being monitored by the PICU nurse?

- Every hour
- Every 4 hours
- Every 6 hours
- Every 8 hours
- Every 12 hours
- Every 24 hours
- Other (please specify)

* 23. What clinical symptoms and vital parameters do you consider important indicators for an excessive fluid state? (please check ALL that apply)

- Increased blood pressure
- Peripheral oedema
- Signs of pulmonary oedema
- Hepatomegaly
- Increase in bodyweight
- Increased CVP
- A positive total fluid balance (intake minus output)
- In need of adjustments in ventilator setting
- Other (please specify)

* 24. What clinical symptoms and vital parameters do you consider important indicators for an excessive fluid state, in need of fluid removal therapy OR fluid restriction? (please check ALL that apply)

- Increased blood pressure
- Peripheral oedema
- Signs of pulmonary oedema
- Hepatomegaly
- Increase in bodyweight
- Increased CVP
- A positive total fluid balance (intake minus output)
- In need of adjustments in ventilator setting
- Other (please specify)

* 25. What tools (both clinical and diagnostic) do you use regularly for diagnosing fluid overload. (please check ALL that apply)

- Lung ultrasound
- Cardiac ultrasound
- Chest X-ray
- PICCO (transpulmonary thermodilution)
- Laboratory values (e.g. urea, creatinine, NT proBNP, etc.)
- Other (please specify)

6. Interventions performed during fluid imbalances.

The following questions refer to interventions used in case of fluid imbalances in (hemodynamic stable) mechanically ventilated PICU patients.

Fill in the answers as it applies for the ‘’general” invasive mechanically ventilated (expected duration of > 48 hrs) PICU patients. *Excluding post-transplant patients, post-cardiothoracic surgery patient and patients with pre-existing cardiac and kidney dysfunction prior to admission to PICU.*

* 26. How do you determine if your patient is in need of fluid resuscitation? (please check ALL that apply)

- [ ] Based upon clinical signs (like refill, colour, peripheral temperature)
- [ ] Based upon heart rate and or blood pressure
- [ ] Based upon urine production
- [ ] Based upon additional diagnostics (ultrasound, advanced hemodynamic monitoring, etc)
- [ ] Other (please specify)
- [ ] Based upon laboratory diagnostics like urea
- [ ] Based upon increased lactate level
- [ ] Based upon a measure of fluid responsiveness

* 27. If case of a hypovolemic state, what volume of fluid bolus do you typically give to a invasive mechanically ventilated child that is hemodynamically stable without cardiac disease?

- ○ None
- ○ 5ml/kg
- ○ 10ml/kg
- ○ 15ml/kg
- ○ 20ml/kg
- ○ >20ml/kg

* 28. Do you determine fluid responsiveness before administering a fluid bolus?

- ○ Always
- ○ Usually
- ○ Sometimes
- ○ Rarely
- ○ Never

* 29. If you determine fluid responsiveness, what method do you use most often? (please check ALL that apply)

- [ ] N/A
- [ ] Passive leg raising
- [ ] Arterial pressure variations
- [ ] Peak flow variations in aorta using ultrasound/doppler
- [ ] Mini fluid challenge
- [ ] CVP
- [ ] Diameter and/or collabsability of the inferior vena cava using ultrasound
- [ ] Liver compression
- [ ] Other (please specify)

* 30. If you deliver a fluid bolus as fluid resuscitation, how do you establish its beneficial effect? (please check ALL that apply)

- [ ] An increase in blood pressure
- [ ] A decrease in heart rate
- [ ] An increase in urine production
- [ ] Other (please specify)
- [ ] An increase in cardiac output
- [ ] Improved clinical signs
- [ ] Improved NIRS measurement

* 31. What cumulative fluid balance (%) (since admission in PICU AND start of mechanical ventilation) is a reason for making fluid management changes?

- ( ) Even fluid balance; one should strive for a negative fluid balance
- ( ) 0 - 5% fluid positive*
- ( ) 5% - 10% fluid positive *
- ( ) Other (please specify)
- ( ) 10% - 15% fluid positive*
- ( ) 15% - 20% fluid positive*

* 32. In case of changing fluid management, due to positive fluid balance or clinical signs of fluid overload, what is the **initial** intervention used for fluid removal? (please check ALL that apply)

- [ ] Lowering fluid maintenance
- [ ] Avoidance of maintenance fluid and minimisation of drug diluents
- [ ] Start diuretic drug therapy
- [ ] Other (please specify)
- [ ] Early start of renal replacement therapy
- [ ] Watch and wait, evaluate later on
- [ ] I do not consider fluid overload to be a problem

* 33. What is the preferred **initial** drug therapy used for fluid removal in case of positive fluid balance or signs of fluid overload in invasive mechanically ventilated PICU patients? (please check ALL that apply)

- [ ] Intermittent loop diuretics
- [ ] Continuous loop diuretics drip infusion
- [ ] Thiazide diuretic (e.g. hydrochlorothiazide)
- [ ] Potassium sparing (e.g. spironolactone)
- [ ] I do not use diuretics for fluid removal therapy
- [ ] Other (please specify)

* 34. How often is continuous renal replacement treatment (CRRT) used to manage fluid overload as the sole indication?

- ◯ Always
- ◯ Usually
- ◯ Sometimes
- ◯ Rarely
- ◯ Never

* 35. What are the criteria (related to fluid overload) to start CRRT? (please check ALL that apply)

- ☐ Oliguria/anuria
- ☐ Metabolic acidosis
- ☐ Hyperkalaemia
- ☐ Other (please specify)
- ☐ Pulmonary oedema
- ☐ Increase serum creatinine/urea
- ☐ Fluid overload >10% unresponsive to diuretics

Survey Total Fluid Management in Invasive Mechanically Ventilated Children

7. Nutrition and enteral feeding

* 36. Is there a paediatric nutrition support team or unit in your institution?

- ◯ Yes
- ◯ No

* 37. Energy requirements (kCal) may be decreased, in order to maintain an acceptable fluid blanace/status in invasive mechanically ventilated children.

- ◯ Strongly agree
- ◯ Agree
- ◯ Neither agree nor disagree
- ◯ Disagree
- ◯ Strongly disagree

* 38. What is, in your opinion the best method of fluid administration in invasive mechanically ventilated patients (non-resuscitation).

- ◯ Enteral
- ◯ Parenteral

* 39. In case of starting enteral feeding in mechanically ventilated patients, do you, if possible, switch from intravenous fluid administration to enteral fluid administration?

- Always
- Usually
- Sometimes
- Rarely
- Never

Survey Total Fluid Management in Invasive Mechanically Ventilated Children

8. Future clinical trial

The following questions will ask about your opinion and your perspectives regarding the design and content of a future clinical trial regarding maintenance fluid management in invasive mechanically ventilated PICU patients.

* 40. Further research in fluid management is essential to improve our understanding and tailoring medical care in invasive mechanically ventilated patients.

- Strongly agree
- Agree
- Neither agree nor disagree
- Disagree
- Strongly disagree

* 41. Would you be willing to include patients in a clinical trial comparing a liberal versus a conservative fluid management in invasive mechanically ventilated patients?

- Yes
- No (please specify)

* 42. In case of participation in a future multicentre randomized trial, what approach would you prefer?

- Cluster (hospital) randomization
- Patient randomization with strict detailed flowsheet including intervention protocol to prevent/treat fluid overload.
- Patient randomization in a pragmatic clinical trial; A target fluid balance and nutritional intake will be provided. The intervention(s) to reach the target fluid balance is up to the attending physician as he or she sees fit.
- Other (please specify)

43. What is, in your opinion, an important primary outcome regarding fluid management therapy in invasive mechanically ventilated PICU patients?

44. Should a future trial include a specific patient category (e.g. only invasive mechanically ventilated patients with respiratory tract infection).

45. Would you have any other suggestions in the design of this future trial that would be of importance?

Survey Total Fluid Management in Invasive Mechanically Ventilated Children

9. End of survey

We would like to thank you for participating in this survey. Your answers will contribute in future studies. We appreciate your willingness to give up your time to help improve our understanding of this issue.

46. Do you have any other questions, comments or concerns?

47. If you would you like to receive a copy of the results of this survey, please leave us your email address. The results will be forwarded to you when the study is completed.

Email Address
